# Immortal-time bias in older vs younger age groups: a simulation study with application to a population-based cohort of patients with colon cancer

**DOI:** 10.1038/s41416-023-02187-0

**Published:** 2023-02-09

**Authors:** Sophie Pilleron, Camille Maringe, Eva J. A. Morris, Clémence Leyrat

**Affiliations:** 1 Nuffield Department of Population Health, University of Oxford, Big Data Institute, Old Road Campus, Oxford, OX3 7LF UK; 2 Ageing, Cancer, and Disparities Research Unit, Department of Precision Health, Luxembourg Institute of Health, 1A-B, rue Thomas Edison, 1445 Strassen, Luxembourg; 3 Inequalities in Cancer Outcomes Network, London School of Hygiene and Tropical Medicine, Keppel Street, London, WC1E 7HT UK; 4 Department of Medical Statistics, Faculty of Epidemiology and Population Health, London School of Hygiene and Tropical Medicine, London, UK

**Keywords:** Cancer epidemiology, Medical research

## Abstract

**Background:**

In observational studies, the risk of immortal-time bias (ITB) increases with the likelihood of early death, itself increasing with age. We investigated how age impacts the magnitude of ITB when estimating the effect of surgery on 1-year overall survival (OS) in patients with Stage IV colon cancer aged 50–74 and 75–84 in England.

**Methods:**

Using simulations, we compared estimates from a time-fixed exposure model to three statistical methods addressing ITB: time-varying exposure, delayed entry and landmark methods. We then estimated the effect of surgery on OS using a population-based cohort of patients from the CORECT-R resource and conducted the analysis using the emulated target trial framework.

**Results:**

In simulations, the magnitude of ITB was larger among older patients when their probability of early death increased or treatment was delayed. The bias was corrected using the methods addressing ITB. When applied to CORECT-R data, these methods yielded a smaller effect of surgery than the time-fixed exposure approach but effects were similar in both age groups.

**Conclusion:**

ITB must be addressed in all longitudinal studies, particularly, when investigating the effect of exposure on an outcome in different groups of people (e.g., age groups) with different distributions of exposure and outcomes.

## Introduction

Immortal-time bias occurs in longitudinal studies when the exposure is defined based on information available after the start of the participants’ follow-up. This is typically the case when the start of follow-up and treatment initiation do not coincide [1]. In cancer literature, a classic example of ITB is when one wants to estimate the effectiveness of a treatment on survival by comparing survival measures (e.g., median survival, overall survival) from cancer diagnosis between patients who do, and do not, receive it. In practice, treatment is rarely initiated on the day of diagnosis, and therefore, in order to initiate the treatment at some point in time, patients must remain alive at least until the time of the treatment receipt; this period is, therefore, called “immortal time”. By defining the study groups based on the observed treatment assigned later, patients who may have been offered the treatment but died before initiation would contribute to the untreated group and as such inflate the number of deaths in that group. In a hypothetical trial in which patients are randomised to treatment groups at the time of diagnosis, patients who die before treatment initiation would be on average equally represented in both study groups, and the ITB would not be a concern. In non-randomised study design, however, patients in the treated group would have an apparent survival advantage compared to those in the non-treated group, regardless of the efficacy of the treatment studied. Any apparent survival benefit in the treatment group may not, therefore, indicate a benefit of the treatment.

Several recent papers in epidemiology from different medical fields (oncology, nephrology, cardiology, etc.) drew attention to this bias [1–5]. However, it seems that this bias is still commonly misunderstood or overlooked in the cancer survival literature. Indeed, ITB was commonly seen in recent literature reviews [6–9]. However, several statistical methods are available to address the issue, including using the treatment as a time-varying exposure, the delayed entry approach or conditioning on a given survival time (landmark time) [4, 10].

Patients aged 75 years or older are underrepresented in randomised clinical trials, and observational studies are often used to study the effectiveness of treatment in terms of survival, with sometimes comparison between older patients and younger patients. Yet, the magnitude of ITB increases with the likelihood of early death, which itself increases as chronological age increases. Therefore, the magnitude of the bias may worsen with age. However, to our knowledge, no studies evaluated the impact of age on the magnitude of the ITB in cancer research and assessed the performance of standard and suitable analysis methods to account for this bias.

This study, therefore, aims to describe how the magnitude of ITB may differ in relation to age when age modifies the risk of death, the likelihood of receiving the treatment, or both. It also investigates the utility of several analytical techniques to account for this bias in practice. Initially, a simulation study was conducted to empirically illustrate the impact of age on the magnitude of ITB under different scenarios when using a time-fixed exposure statistical approach prone to ITB, and to compare the performance of three alternative methods to account for this bias. Then, these methods were applied to estimate the effect of surgery performed within 6 months of diagnosis on 1-year overall survival in patients diagnosed with Stage IV colon cancer aged 50–74 and separately in those aged 75–84 in England using data from the CORECT-R resource [11].

### The problem

To estimate the effect of surgery performed within 6 months of diagnosis on the 1-year overall survival probability from cancer diagnosis in those who do, and do not, receive the treatment and by age group one can use the Kaplan–Meier estimator or a Cox regression model by simply including surgery status in the model, that is, whether the patients received surgery in the 6 months following the diagnosis or not. This considers treatment as a time-fixed exposure. These methods wrongly assume that surgery occurs at cancer diagnosis whereas in practice, this may not be the case.

To illustrate the risk of ITB obtained using standard statistical approaches, we generated simplified data based on our illustrative example, for the estimation of the effect of surgery within 6 months on 1-year overall survival in patients diagnosed with Stage IV colon cancer aged 50–74 (called younger patients hereafter) and separately, in those aged 75–84 (older patients hereafter). Data were generated under four scenarios in which the probability and timing of death and the probability and timing of treatment could differ between age groups. For each scenario, we generated data following Weibull distributions for 100,000 patients, 50,000 in the younger group, and 50,000 in the older group (details below). For illustrative purposes, the data were generated under the hypothesis of no treatment effect. Then, for each patient, the surgical treatment status and time to surgery, and vital status and survival time were independently generated from Weibull distributions, with chosen parameters to correspond to the following scenarios:

- Scenario 1: younger and older patients have the same survival and treatment distributions.
- Scenario 2: the probability of having surgery within 6 months and the distribution of time to surgery, as well as the 1-year survival probabilities are the same for the two age groups, but deaths occurred earlier among older patients.
- Scenario 3: the 1-year survival probabilities and survival times, as well as the probability of having received surgery at 6 months, are the same in the two age groups, but older patients are treated later on average.
- Scenario 4: the 1-year survival probability and the probability of receiving surgery within 6 months are lower among older patients than among younger patients, and older patients die sooner and receive treatment later than younger patients.

**Fig. 1:**
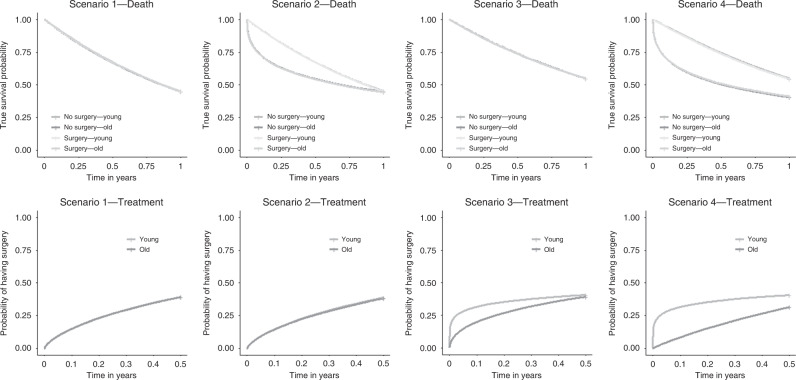
Scenarios used in simulations.

We used different shape parameters to mimic different timing of events while keeping the mortality rate roughly constant. This was done in order to illustrate that ITB may be worse in studies among older people in the plausible situation in which they die more quickly than younger patients, whatever the treatment status, and they tend to undergo surgery a bit later. We started with a scenario where the timing of death and treatment was the same in both age groups and then we varied each timing at a time to move towards scenarios likely to occur in practice (such as Scenario 4). The generated treatment status and time to treatment correspond to the intention to treat, that is, to what would have been observed if the patients could not die. However, for patients whose generated survival time was shorter than their generated time to surgery, their observed treatment status was set to 0. These scenarios are depicted in Fig. 1.

**Fig. 2:**
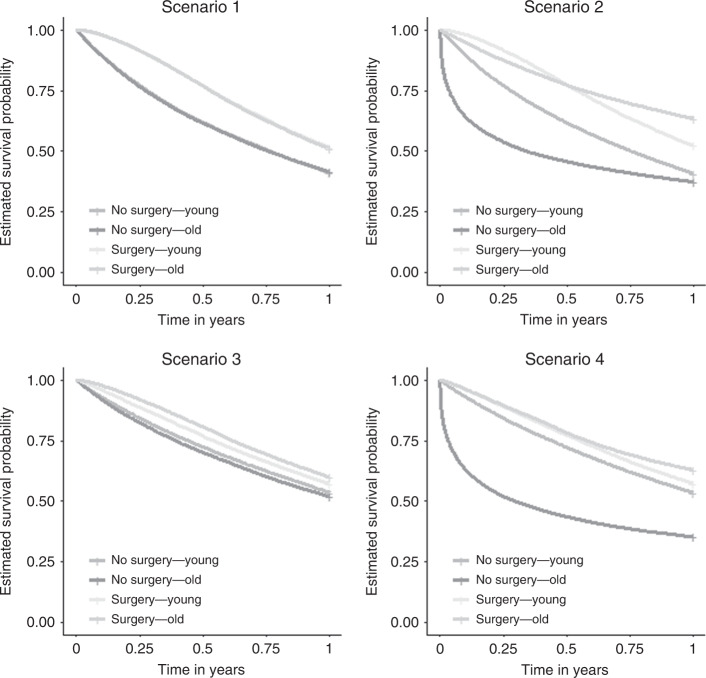
Overall survival estimated using the time-fixed exposure method based on the four scenarios in young and old groups.

Then 1-year overall survival in both treatment groups was estimated separately for younger and older patients, using a Cox regression model including treatment as a time-fixed exposure (Fig. 2).

In Scenario 1, surgery as a time-fixed treatment led to a substantial bias due to immortal time: while the true treatment effect is 0 in both age groups, the observed differences in 1-year survival probabilities were about 10% in both age groups. However, because survival and treatment distributions were identical among younger and older patients, the magnitude of the ITB was the same in the two groups. In Scenarios 2 and 3, the magnitude of ITB was larger among older patients. In both cases, this is because the number of patients who died before receiving surgery was larger among older patients. This phenomenon was further amplified in Scenario 4, in which older patients had a higher probability of death (Fig. 2).

This illustrates the need for appropriate methods to obtain treatment effect estimates not affected by immortal-time bias.

### Methods for handling ITB

#### Statistical methods

Several statistical methods have been proposed to address the ITB. One of them is to move forward the time 0 and set up a new time 0 (landmark time) at a time where, for instance, most patients are likely to have received treatment, such as 6 months after diagnosis (researchers should define this period a priori for their research question based on clinical and field knowledge rather than data) [12, 13]. All patients who died before the landmark time are excluded from the analysis. All patients who do receive treatment after the landmark time are categorised in the untreated group. This method is known as landmark analysis. It addresses the issue of ITB by conditioning on survival time. The overall survival from diagnosis but among patients who survived at least until the landmark time can then be estimated using standard statistical methods, such as using Kaplan–Meier estimator or a Cox regression model. Because of the exclusion of all deaths occurring before the landmark time, this method estimates the effect of treatment in patients who were alive at the landmark time, therefore this method estimates a conditional effect, and it cannot be interpreted as the marginal effect of surgery in the population.

Another technique considers patients as untreated until they are treated and treated thereafter. The patient’s treatment status therefore changes over time. This method is called time-varying exposure analysis [14]. Time-varying exposure analysis, such as the Cox model with treatment as a time-varying variable, can be used to compare overall survival from diagnosis in both treated and untreated groups. Contrary to the landmark analysis, the whole sample is analysed, which allows the estimation of the marginal treatment effect from diagnosis on the entire population.

A third technique to handle immortal-time bias is to allow for delayed entry. This method is essentially similar to the time-varying exposure method described previously, except that two models estimate separately the survival probabilities among the treated and the untreated. The model under no treatment includes the entire follow-up of the untreated patients, and the time between the diagnosis and the treatment for the treated patients. The model under treatment includes patients from the time of treatment to the end of follow-up. By having two models, this approach would be equivalent to the time-varying exposure approach in which all the covariate treatment interactions would be included. However, a drawback is that unlike the time-varying exposure and landmark analyses, model-based standard errors cannot be obtained because there are two independent models involved, therefore, standard errors must be obtained using non-parametric bootstrap.

#### Emulated target trial framework

Emulating a target trial is another way to handle ITB [15]. Selection of patients, definitions of exposures, outcomes and causal estimand are done to mimic as closely as possible the “ideal” randomised clinical trial that we would like—but cannot—conduct. Then, a specific statistical method has been proposed to handle ITB in this framework. The method consists of (i) cloning, (ii) censoring and (iii) weighting participants to address (i) confounding at baseline and (ii) ITB, and (iii) informative censoring introduced in step (ii). This approach makes the design of observational studies more transparent and allows the estimation of the effect of the intent to perform surgery.

## Methods

### Simulation study

#### Aims

The aim of the simulation study was to investigate under which circumstances the magnitude of the ITB differs between younger and older populations and to illustrate the performance of the time-varying approach, the delayed entry method and the landmark analysis for the estimation of treatment effects in the presence of ITB. The simulation study was conducted, therefore, to investigate the impact of age on ITB when no other biases are in play. This simulation is illustrative and does not aim to fully evaluate the statistical properties of the aforementioned methods.

#### Data generation

The data were generated as described previously in “The problem”, except that the sample size was 1000 for each generated dataset. Supplementary Table S1 shows the values of the shape and scale parameters of the Weibull distributions in each scenario. In all the scenarios, no treatment effect in either age group was assumed. All the simulations were conducted in R 3.6.0 [16]. We used the R package *simsurv* to generate the time-to-event data [17].

#### Estimand

Our estimand of interest was the difference in 1-year survival probabilities following diagnosis between treated and untreated patients, among younger and older patients separately.

#### Methods

In each generated dataset, we estimated the difference in 1-year overall survival probabilities using a Cox regression model based on the time-fixed exposure approach as well as the three approaches addressing the ITB, which are landmark analysis, time-varying exposure analysis, and delayed entry method. Given that the emulated trial approach with cloning, censoring and weighting is computationally intensive, we did not include it in our simulation. The models were estimated separately by age group. For the time-varying Cox model, the data were split at each time a death occurs, meaning that a row was one patient per time interval. Non-parametric bootstrap was used to estimate the 95% confidence intervals for the difference in survival probabilities. Normal-based confidence intervals were constructed using 5000 replications.

#### Performance measure

We estimated the bias of the estimate of the difference in 1-year survival probabilities, as well as the average bootstrap standard errors across simulations and empirical standard error.

### Illustrative example

We included 10,392 patients aged 50–74 and 6562 patients aged 75–84 years old diagnosed with Stage IV colon cancer (ICD-10 code: C18) in England between 2014 and 2017 from the COloRECTal cancer Repository (CORECT-R). The CORECT-R is a national population-based resource that provides information about all patients diagnosed with colorectal cancer in England thanks to the linkage of cancer registry data to a variety of datasets including hospitalisation data from Hospital Episode Statistics (HES) [11]. We sequentially excluded patients with unknown vital status (*n* = 15), unknown survival time (*n* = 11), patients with no record in HES (*n* = 282), unknown ethnicity (*n* = 787) and those whose surgery was before the cancer diagnosis (*n* = 370). As we are interested in assessing the effectiveness of major resection against no surgery, we further excluded patients who had minor resection, stoma, stent, or bypass (*n* = 2331), leaving 13,158 patients for analysis. All patients were followed up to their death, or December 31, 2018, whichever comes first. We censored all patients alive beyond 1 year after diagnosis. Patients who underwent surgery later than 6 months after diagnosis were considered untreated. We estimated 1-year OS in both age groups (i.e., 50–74 and 75–84) by surgery status (yes/no) using the time-fixed exposure method and the four methods accounting for ITB introduced previously. For the landmark analysis, we chose a landmark time at 6 months after diagnosis, most surgeries occurring within this time window. We excluded all patients who died before the landmark time. For the time-varying exposure approach, we ran a Cox regression model including treatment as a time-varying variable. For the delayed entry method, we fitted two separate Cox regression models in all untreated patients (including patients treated but censored at the time of surgery) and treated patients, respectively.

For the emulated target trial approach, we followed the recommendations in Maringe et al. [15]. First, we specified the target trial we want to emulate in Supplementary Table S3. Second, we created two copies of each observation, each allocated to a different arm. Third, we censored patient follow-up times when their treatment was no longer compatible with the arm they were in and defined outcomes and survival time. Fourth, to account for informative censoring due to artificial censoring, we estimated weights by predicting the individual probabilities of remaining uncensored at each time of event using a Cox regression model adjusted for confounders identified using a directed acyclic graph (Supplementary Fig. S1). Finally, we fitted a weighted Cox regression model in each arm.

The analysis of the DAG shown in Supplementary Fig. S1 identified that the following confounding variables required adjustment: chronological age, sex, ethnicity, socio-economic circumstances, socio-deprivation residential area, comorbidities, ethnicity, physiological age, social support. Ethnicity, socio-economic circumstances, physiological age and social support were not observed and are potential sources of unobserved confounding. Consequently, all models were adjusted for age (restricted cubic spline with 1 interior knot placed at the median of the observed distribution of age, and two boundary knots placed at 10% and 90% quantile of the observed distribution of age), sex, socio-economic deprivation categorised into fifths, ethnicity categorised into White and non-White due to small numbers and Charlson’s comorbidity index categorised into 0, 1–2, 3+. We then estimated the marginal overall survival in the treated and untreated groups by predicting the individual 1-year survival for all patients under two hypothetical scenarios: as if all the population was treated and then as if the whole population was untreated. The effect of the surgery was the difference in mean 1-year overall survival in these two hypothetical scenarios. Non-parametric bootstrap, using 1000 replications was used to calculate standard errors and derive 95% normal-based confidence intervals.

We performed statistical analyses using R statistical software (version 4.2.1; R Development Core Team, 2021).

## Results

### Simulation study

**Fig. 3:**
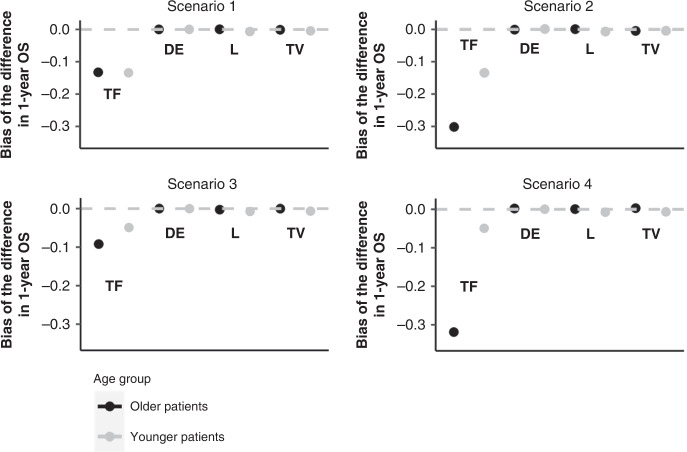
Bias of the difference in 1-year overall survival (OS) based on the four scenarios. TF time-fixed exposure method, DE delayed entry method, L landmark method, TV time-varying exposure method. Note: a difference of 0 indicates there is no immortal-time bias.

The results of the four scenarios are presented in Fig. 3. As already illustrated in “The problem”, using a time-fixed exposure leads to a strong bias in all the scenarios (bias ranging from −0.092 in Scenario 3 to −0.319 in Scenario 4). As expected, if there are no differences in terms of survival and probability and timing of treatment between age groups, the magnitude of the ITB is similar in the two age groups (Scenario 1). However, the magnitude of the ITB increases with earlier death and delayed treatment (Scenarios 2 to 4), which are likely to be more common among older patients in comparison to younger patients. With a time-varying exposure, a delayed entry model and landmark analysis, the bias was corrected. None of these approaches led to convergence issues (convergence rate of 100% across scenarios). Out of the three unbiased approaches, the delayed entry methods led to the smallest empirical standard error across simulations (Supplementary Table S2).

### Illustrative example

Table 1 presents patients’ characteristics by age group. Median follow-up time was shorter in older patients (141 days) as compared to younger patients (360 days). Under a third of older patients underwent surgery against 46% of younger patients. A vast majority of surgeries occurred within 6 months from diagnosis in both groups. There were more women in the older age group, and older patients had a higher Charlson’s comorbidity index than younger patients. Deprivation level was similarly distributed in both age groups.

**Table 1:** Characteristics of our study sample by age group.

	50–74 years old	75–84 years old
*n*	7984	5174
Number of deaths within the first year after diagnosis (%)	4031 (50.5)	3715 (71.8)
Number of deaths within the first 6 months after diagnosis (%)	2890 (36.2)	2880 (55.7)
Median follow-up time in days (IQR)	360 (86–365)	141 (46–365)
Surgery, *n* (%)	3670 (46.0)	1581 (30.6)
Surgery within 6 months, *n* (% of all patients with surgery)	3341 (91.0)	1530 (96.8)
Median age in years (IQR)	66 (60–70)	79 (77–82)
Females, *n* (%)	3624 (45.4)	2428 (46.9)
Deprivation index quintiles, *n* (%)		
1—Less deprived	1679 (21.0)	1124 (21.7)
2	1754 (22.0)	1210 (23.4)
3	1608 (20.1)	1105 (21.4)
4	1501 (18.8)	940 (18.2)
5—Most deprived	1442 (18.1)	795 (15.4)
Charlson’s comorbidity index, *n* (%)		
0	5747 (72.0)	2825 (54.6)
1–2	1723 (21.6)	1619 (31.3)
3+	514 (6.4)	730 (14.1)

*IQR* interquartile range.

Table 2 presents 1-year OS estimates in each treatment group and the difference in survival between the two treatment groups by age category and analysis method.

**Table 2:** One-year overall survival (OS - %) by age category, receipt of surgery and method to address immortal-time bias (reference group: no surgery group).

		50–74 years old			75–84 years old		
		No surgery	Surgery		No surgery	Surgery	
Method	Estimand	1-year OS (95% confidence interval)	1-year OS (95% confidence interval)	**Difference in 1-year survival**	1-year OS (95% confidence interval)	1-year OS (95% confidence interval)	**Difference in 1-year survival**
Time-fixed exposure method	1-year overall survival	32.7 (31.3; 34.1)	71.9 (70.4; 73.4)	−39.2 (−41.2; −37.2)	15.4 (14.2; 16.6)	57.4 (55.0; 59.8)	−42.1 (−44.8; −39.4)
Landmark analysis	1-year overall survival conditioning on being alive beyond landmark time (i.e., 6 months)	69.8 (67.9; 71.7)	83.9 (82.5; 85.3)	−14.1 (−16.5; −11.7)	52.0 (49.1; 54.9)	75.3 (72.9; 77.7)	−23.2 (−27.1; −19.3)
Time-varying exposure	1-year overall survival	36.8 (35.4; 38.2)	68.5 (66.9; 70 .1)	−31.7 (−33.8; −29.6)	17.5 (16.3; 18.7)	53.4 (50.9; 55.9)	−35.9 (−38.7; −33.1)
Delayed entry method	1-year overall survival	37.8 (36.4; 39.2)	68.4 (66.6; 70.2)	−30.6 (−32.9; −28.3)	18.1 (16.8; 19.4)	53.4 (50.6; 56.2)	−35.3 (−38.4; −32.2)
Emulated target trial framework	1-year overall survival	37.8 (36.4; 39.3)	54.4 (53.6; 55.3)	−16.6 (−18.0; −15.2)	18.0 (16.8; 19.3)	33.6 (32.5; 34.8)	−15.6 (−17.1; −14.2)

When compared to both the time-varying exposure and the delayed entry approaches, the time-fixed exposure approach led to a larger effect of surgery on 1-year OS, than other methods, as expected. Both the time-varying exposure and delayed entry methods provided similar estimates of the effect of surgery on 1-year OS in both age groups (31% points in the younger age group and 35% points in the older age group). Besides, the difference in the effect of surgery between the time-fixed exposure approach and the time-varying exposure or delayed entry method is about 6–8 points suggesting no difference in the magnitude of the ITB across age groups.

Using the landmark analysis, survival estimates were higher than those estimated using the other methods due to the exclusion of patients who died within the first 6 months after diagnosis. Given that a different sample is used for this analysis, a direct comparison of surgery effect estimates with other approaches is not possible. In patients who survived at least 6 months, the effect of surgery on the conditional 1-year OS was higher in the 75–84 age group (−23 point difference; 95% confidence interval: −27; −19) than in the 50–74 age group (−14 point difference: −17; −12).

Using the emulated target trial framework, the effect of surgery on 1-year survival was similar in both age groups (about 16 point difference) and, as expected, smaller than those estimated using both the time-varying exposure and the delayed entry methods because the targeted estimand is different.

## Discussion

In a context where older patients with cancer are still less likely to be included in randomised clinical trials [18], and more generally where randomised clinical trials are deemed unfeasible or unethical in some circumstances (e.g., resection of colorectal cancer liver and lung metastases [19]), population-based observational data (e.g., cancer registry, hospital statistics) are a good source of information that may be used for comparative effectiveness research. However, observational studies are prone to a certain number of biases, including ITB, and researchers have to be careful when planning their study. We show the magnitude of the ITB varies based on the distribution of death and patterns of treatment receipts over time. We provide R scripts for relevant statistical methods that correct for the ITB.

We presented four methods that were proposed to control for the ITB: the landmark analysis, the analysis considering treatment as a time-varying exposure, the delayed entry method, and the emulated target trial framework. The first two methods are the most commonly used in the literature. Both the time-varying exposure and the delayed entry method estimate the effect of treatment on overall survival from cancer diagnosis (such as surgery on 1-year survival in our study), while the landmark analysis estimates survival probabilities conditionally to surviving beyond a pre-defined landmark time (i.e., 6 months in our study). Consequently, the resulting point estimates cannot be compared with the other two statistical approaches. The quantity to estimate will depend on the question asked: are we interested in the effect of surgery on survival beyond 1 year from diagnosis or are we interested in knowing the effect of surgery beyond 1 year since diagnosis if patients survive for at least a certain period of time after diagnosis, for instance because of high early mortality prior to treatment?

The landmark analysis is easy to implement and to interpret, but this method excludes patients who died before the landmark time. Therefore, the results depend on the choice of the landmark time. This method may provide useful information about survival prognosis for the clinical management of patients. Time-varying exposure analysis and the delayed entry method use the whole sample of patients for analysis. The delayed entry method is similar to the time-varying exposure method with the difference this approach requests to model the estimator of interest separately in the treated and the untreated groups. Therefore, it allows the effect of covariates to differ by treatment group.

The emulated target trial framework is gaining in popularity and may be established in the near future as a reference approach to study the effect of a treatment on an outcome using observational data. Borrowing from the randomised clinical trial structure and definitions, this approach forces researchers to clearly state the research question, and define the exposure, outcome and the effect to estimate, as well as highlight caveats and biases from the data. However, this framework estimates the effect of surgery from diagnosis, at the time at which surgery might be indicated while other methods estimate the effect of surgery once received, precluding direct comparisons of estimates between all methods.

In our illustrative example, the effect of the surgery was similar across age groups and across methods (excluding landmark analysis) suggesting the magnitude of ITB was similar across group (same difference between estimates obtained using fixed-time exposure method and varying-time exposure or delayed entry methods across age groups). This contrasts with the results of our simulation study which showed how the probability of receiving surgery, and the probability of death influenced the magnitude of the ITB. One hypothesis is that older patients may die sooner and receive surgery earlier than younger patients, cancelling the respective effects on ITB. Because we used observational data, we cannot rule out residual confounding due to unmeasured confounding factors. Indeed, we regret the lack of information about patients’ fitness, geriatric conditions, social support, or care providers which play the role of confounder in the relationship between surgery and survival. Also, we did not make the distinction between emergency surgery and elective surgery. Older patients are more likely to be diagnosed through an emergency presentation which is associated with poor survival prospects [20]. The greater effect of surgery in older patients than in younger adults observed using the landmark analysis may be explained by the selection of the fittest patients as they had to be alive 6 months after diagnosis to be included in the analysis. Finally, we included patients with known Stage IV colon cancer while 9.8% have an unknown stage, which may have led to selection bias. The stage at diagnosis is more likely to be missing in older adults than younger adults [21, 22]. Further studies on the effect of surgery on survival in patients diagnosed with colon cancer is, therefore, warranted.

## Conclusion

Immortal-time bias is often overlooked in longitudinal studies using observational data, but it is important to consider when the inclusion of participants/patients in the study does not coincide with their allocation to one of the groups being compared, as for instance, in survival analysis when comparing survival between groups defined after the start of follow-up of participants/patients (e.g., treatment). This is even more important when investigating the effect of exposure on an outcome in different groups of individuals who may have different probabilities distributions for exposure or outcomes as in our example, where the likelihoods of early death and treatment receipt are not evenly distributed across all age groups. In all circumstances, researchers have to plan carefully their study and their analysis. For further reading on the topic, we encourage interested readers to read [1, 15, 23, 24].

## Supplementary information

Supplemental Material 1 - Supplemental Table

Supplemental Material 2 - Simulation code

Supplemental Material 3 - CORECTR Analysis code

Supplemental Material 4 - Emulated Target Trial Analysis code

## Data Availability

CORECT-R data are available via application, see https://www.ndph.ox.ac.uk/corectr/corect-r.

## Code availability

The R codes for the simulation study, the CORECT-R analysis, and the emulated target trial analysis are available in Supplemental Files 2, 3 and 4, respectively.
