# Supplementary material for: Immortal-time bias in older vs younger age groups: a simulation study with application to a population-based cohort of patients with colon cancer
Source: Br J Cancer. 2023 Feb 9;128(8):1521–8. doi: 10.1038/s41416-023-02187-0 (PMC10070415; doi:10.1038/s41416-023-02187-0)
Supplement: Supplementary file 1 — Supplemental Material 1 - Supplemental Table [file 41416_2023_2187_MOESM1_ESM.docx]

**Supplement material 1**

**Immortal time bias in older vs younger age groups: a simulation study with application to a population-based cohort of patients with colon cancer**

Sophie Pilleron, Camille Maringe, Eva JA Morris, Clémence Leyrat

**List of Tables**

Table S1 - Values of the shape and scale parameters of the Weibull distributions for the 7 scenarios

Table S2 – Difference in one-year overall survival based on the different methods and scenarios and its associated standard error (SE)

Table S3 - Specification and emulation of a target trial of surgery versus no treatment within 6 months of diagnosis in patients aged 50-99 years old diagnosed with stage IV colon cancer in England between 2014 and 2017.

**List of Figures**

Figure S – Directed acyclic graph representing the relationship between receipt of diagnosis within 6 months of diagnosis and one-year overall survival in patients with colon cancer in England

**Table S1** - Values of the shape and scale parameters of the Weibull distributions for the 7 scenarios

| **Scenario** | **Treatment model** | | | | **Survival model** | | | |
| --- | --- | --- | --- | --- | --- | --- | --- | --- |
|  | **Younger patients** | | **Older patients** | | **Younger patients** | | **Older patients** | |
|  | **Scale** | **Shape** | **Scale** | **Shape** | **Scale** | **Shape** | **Scale** | **Shape** |
| 1 | 0.8 | 0.7 | 0.8 | 0.7 | 0.8 | 1 | 0.8 | 1 |
| 2 | 0.8 | 0.7 | 0.8 | 0.7 | 0.8 | 1 | 0.8 | 0.4 |
| 3 | 0.6 | 0.2 | 0.7 | 0.5 | 0.6 | 1 | 0.6 | 1 |
| 4 | 0.6 | 0.2 | 0.7 | 0.9 | 0.6 | 1 | 0.9 | 0.4 |

**Table S2** – Difference in one-year overall survival based on the different methods and scenarios and its associated standard error (SE)

|  | Time-fixed Cox Model | | | | Landmark analysis | | | | Time-varying Cox model | | | | Delayed entry method | | | |
| --- | --- | --- | --- | --- | --- | --- | --- | --- | --- | --- | --- | --- | --- | --- | --- | --- |
| Scenario | Older patients | | Younger patients | | Older patients | | Younger patients | | Older patients | | Younger patients | | Older patients | | Younger patients | |
|  | Estimate* | Empirical SE** | Estimate | Empirical SE | Estimate | Empirical SE | Estimate | Empirical SE | Estimate | Empirical SE | Estimate | Empirical SE | Estimate | Empirical SE | Estimate | Empirical SE |
| 1 | -0.133 | 0.045 | -0.134 | 0.043 | 0.000 | 0.048 | -0.007 | 0.054 | -0.001 | 0.049 | -0.005 | 0.047 | 0.000 | 0.007 | 0.000 | 0.009 |
| 2 | -0.302 | 0.045 | -0.134 | 0.043 | 0.001 | 0.046 | -0.007 | 0.054 | -0.005 | 0.061 | -0.004 | 0.047 | -0.001 | 0.005 | 0.001 | 0.007 |
| 3 | -0.092 | 0.044 | -0.049 | 0.041 | -0.003 | 0.042 | -0.007 | 0.047 | 0.000 | 0.047 | -0.006 | 0.042 | 0.000 | 0.009 | 0.000 | 0.011 |
| 4 | -0.319 | 0.047 | -0.049 | 0.041 | 0.001 | 0.053 | -0.007 | 0.047 | 0.003 | 0.063 | -0.006 | 0.042 | 0.002 | 0.005 | 0.000 | 0.008 |
|  |  |  |  |  |  |  |  |  |  |  |  |  |  |  |  |  |
| * Average estimated difference in 1-year overall survival probabilities across the 1000 simulations. The true value is 0. | | | | | |  |  |  |  |  |  |  |  |  |  |  |
| ** Standard deviation of the 1000 estimated difference in 1-year overall survival probabilities | | | | |  |  |  |  |  |  |  |  |  |  |  |  |

Table S3 - Specification and emulation of a target trial of surgery versus no treatment within 6 months of diagnosis in patients aged 50-84 years old diagnosed with stage IV colon cancer in England between 2014 and 2017.

| **Component** |  | **Target trial** |  | **Emulated trial using CORECT-R data** |
| --- | --- | --- | --- | --- |
| **Design** |  | Multicentre open-label two-parallel arm superiority randomised trial. |  |  |
| **Aim** |  | Estimate the effect of receiving major resection within six months of a stage IV colon cancer diagnosis on 1-year overall survival |  | Same |
| **Eligibility** |  | Patients aged 50-84 years old diagnosed with stage IV colon cancer in England between 2014 and 2017 |  | Same |
| **Exclusions** |  | Patients with surgery in the month prior to diagnosis |  | Same |
| **Treatment strategies** |  | 1. Major surgery within six months of diagnosis 2. No surgery in the six months after diagnosis |  | Same |
| **Treatment assignment** |  | Patients are randomly assigned to either strategy |  | Patients are non-randomly assigned to a treatment strategy. Randomisation is emulated via cloning of patients in both arms. |
| **Treatment implementation** |  | None |  | 6 months grace period |
| **Outcome** |  | Death from all causes within a year of diagnosis |  | Same |
| **Type of outcome** |  | Failure time |  | Same |
| **Follow up** |  | Follow up starts at diagnosis, equivalent to treatment assignment |  | Follow up starts at diagnosis, which does not correspond to treatment assignment |
| **Censoring** |  | Loss to follow up |  | Loss to follow up, administrative censoring |
| **Adjustment variables** |  | Age at diagnosis, sex, deprivation levels categorized into fifths, Charlson’s comorbidity index categorised into 0,1-2, 3+ |  | Same |
| **Causal contrast** |  | *Per protocol and intention to treat* |  | *Per protocol*: we do not know what the intention to treat was from the data; In each arm of the emulated trial, patients who deviate from the protocol are censored at their time of deviation |
| **Estimand** |  | Differences in mean one-year survival between arms |  | Same |

*
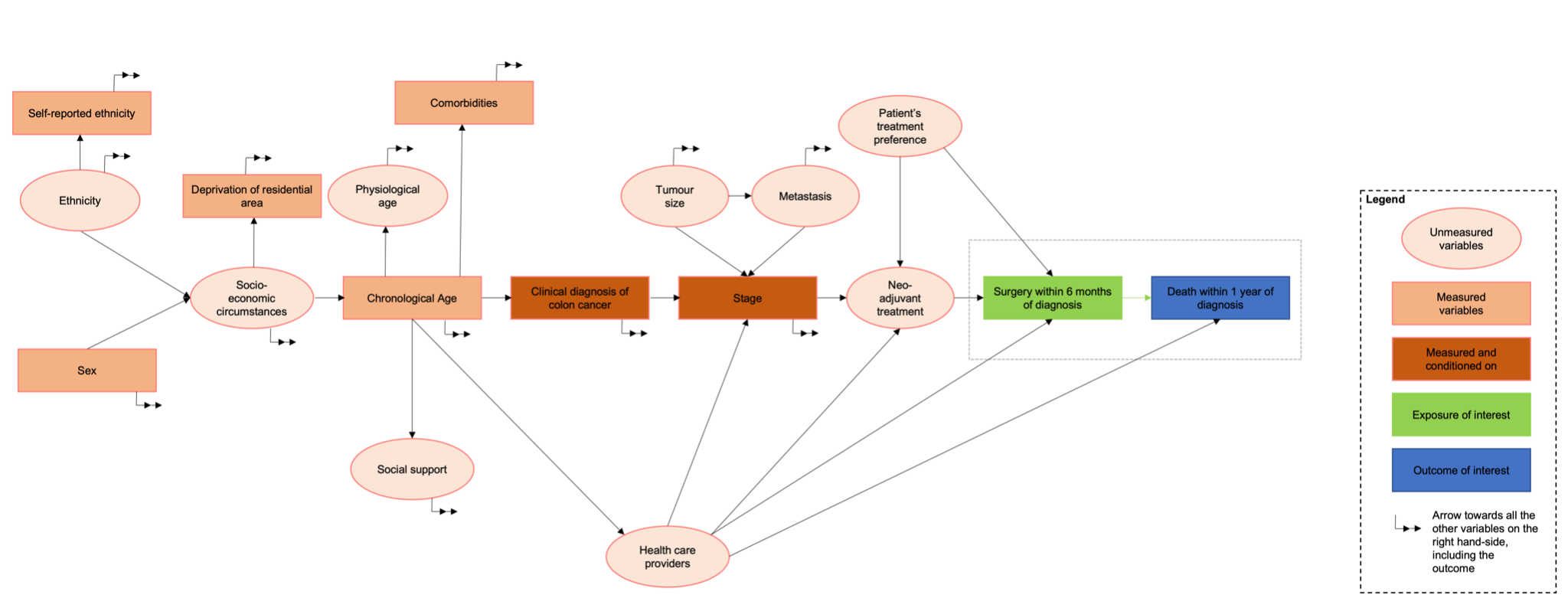
*

**Figure S1** - Directed acyclic graph (DAG) depicting the focal relation between surgery within 6 months of diagnosis and all cause mortality within 1 year of diagnosis. The darker orange nodes “*clinical diagnosis of colon cancer*” and “*stage*” are presented to show that the study is restricted to (or conditioned on) patients diagnosed with stage IV colon cancer.

The minimum set of adjustment is: chronological age, sex, ethnicity, self-reported ethnicity, socio-economic circumstances, socio-deprivation residential area, comorbidities, physiological age, social support. However, ethnicity, socio-economic circumstances, physiological age, social support are not observed and are potential sources of unobserved confounding.
